# Perceived adherence and associated barriers to the national atopic dermatitis guideline: A survey among general practitioners

**DOI:** 10.1080/13814788.2023.2242583

**Published:** 2023-08-21

**Authors:** Aviël Ragamin, Karlijn F. van Halewijn, Marie L.A. Schuttelaar, Marjolein Lugtenberg, Suzanne G.M.A. Pasmans, Gijs Elshout, Renske Schappin

**Affiliations:** a Department of Dermatology, Erasmus MC, University Medical Center, Rotterdam, The Netherlands; b Department of Dermatology, Center of Pediatric Dermatology, Sophia Children’s Hospital, Erasmus MC University Medical Center Rotterdam-Sophia Children’s Hospital, Rotterdam, The Netherlands; c Department of General Practice, Erasmus MC, University Medical Center, Rotterdam, The Netherlands; d Department of Dermatology, University Medical Center Groningen, University of Groningen, Groningen, The Netherlands

**Keywords:** Atopic dermatitis, guideline adherence, general practitioner, primary care

## Abstract

**Background:**

General practitioners (GPs) have an important role in managing patients with atopic dermatitis (AD). Although pivotal, adherence to dermatological guidelines in general practice has not been assessed.

**Objectives:**

To assess GPs’ perceived adherence and barriers to the Dutch AD guideline.

**Methods:**

A survey was conducted among 391 GPs in the Netherlands between December 2021 and May 2022. GPs rated their perceived adherence and perceived barriers concerning five key recommendations of the AD guideline, following an existing framework. The correlation between perceived adherence and barriers was investigated using Spearman’s rank correlation.

**Results:**

A total of 213 GPs (54%) participated. Perceived adherence rates varied across recommendations (43.7% to 98.1%). Lowest adherence was reported for recommendations concerning topical corticosteroids (TCS). Across all recommendations, patient factors (65.6%; SD 11.6) and lack of applicability to specific patient groups (29.5%; SD 10.5) were reported most frequently as barriers. The overall correlation between adherence and barriers was strongest for knowledge (ρ .55; SD .10) and attitude-related factors (range: ρ .40--.62).

**Conclusion:**

GPs’ perceived adherence and barriers vary substantially across recommendations of the AD guideline. In particular, GPs reported lower adherence to recommendations concerning TCS. Next to patient-related factors, strong correlations between adherence perceived by GPs and knowledge and attitude-related barriers suggest the importance of addressing these factors as well to improve adherence.

KEY MESSAGES

- Perceived adherence of general practitioners and associated barriers vary across atopic dermatitis guideline recommendations.
- Next to patient-related barriers, we found strong correlations between adherence and knowledge and attitude-related barriers.
- These insights can improve guideline adherence and care for patients with atopic dermatitis.

## Introduction

Atopic dermatitis (AD) is a common chronic inflammatory skin disease associated with pruritus and recurrent skin lesions [1]. With a prevalence of up to 20% in children and 10% in adults, AD is the skin disorder with the highest total disease burden [2]. Most patients can be treated with emollients and topical corticosteroids (TCS) [3,4]. Less than 10% of all patients with AD need specialist care [3,5]. This underlines the importance of the role of general practitioners (GPs) in the management of patients with AD.

To support GPs, (inter)national clinical practice guidelines have been developed [6,7]. However, many patients do not receive care as clinical guidelines recommend [8]. This problem is multifactorial but guideline adherence by professionals is an essential factor [9,10].

Previous studies investigating overall guideline adherence in the Netherlands reported adherence rates between 66% and 77% [10,11]. Adherence to clinical guidelines and recommendations for skin diseases has not been studied. Compared to other clinical guidelines, adherence of GPs to dermatological guidelines, particularly the AD guideline, may be lower for several reasons. First, GPs receive limited training in skin diseases, which could lead to less knowledge and experience [12]. Additionally, many GPs experience some degree of anxiety about topical corticosteroids, known as corticophobia [13]. This could be enhanced by fear of TCS expressed by patients with AD and their caregivers [13,14].

It would therefore be interesting to investigate GPs’ adherence to the national AD guideline. Moreover, insight into underlying barriers to non-adherence can help to develop strategies to support GPs in caring for patients with AD.

Our study aims to assess Dutch GPs’ perceived adherence and associated barriers to the national AD guideline, which is embedded in the national eczema guideline [6].

## Methods

### Setting

In the Netherlands, the GP is central to primary care and is a gatekeeper to specialist care. Clinical guidelines for GPs are developed by the Dutch College of General Practitioners (NHG). More information on developing and implementing guidelines in the Netherlands can be found in the supplement (S1). All Dutch citizens are required by law to have health insurance. Patients do not pay additional costs for GP-related consultations; GP care is therefore highly accessible. However, for all other care, including medication, citizens must pay an excess of up to a fixed amount (€385,- in 2023).

### Study design

We conducted an electronic quantitative survey between December 2021 and April 2022 among all GPs collaborating for research, internships and GP training within the Department of General Practice of the Erasmus MC University Medical Centre Rotterdam (*N* = 391). After four weeks GPs received a reminder.

### Survey

First, a panel comprised two dermatologists, a GP, two physicians/PhD candidates in AD and two psychologists with a background in guideline adherence research (Table S1). During several meetings, the panel achieved unanimous consensus on selecting 5 key recommendations of the GP guideline for AD, Table 1 [6]. Independent GPs (*n* = 5) and a representative of the national patient organisation for AD were invited to check whether these recommendations accurately reflected the AD guideline. After their approval, a questionnaire based on the framework of Cabana et al. and a questionnaire based on the framework of Lugtenberg et al. was developed [10,15,16].

**Table 1.** Overview of key recommendations.

Item	Key recommendation
1	Emollients are the basis of treatment and should be advised even when the eczema is calm.
2	In severe eczema, starting (briefly) with a class 3 ‘potent’ topical corticosteroid (rather than a class 1 ‘mild’’ or 2 ‘moderate’ topical corticosteroid) is preferred at all ages.
3	For all types of eczema, evaluate the effect of treatment after 1-2 weeks
4	Take a comprehensive medical history in which you always ask for: Onset and course Localisation Nuisance Previous episodes of eczema Influencing factors Treatment
5	Provide oral and written instruction on the application of topical corticosteroids for optimal effect. Instruct patients to use the fingertip-unit method (FTU): a dash of ointment the length of an adult’s fingertip, 1 FTU, corresponds to approximately 0.5 grams of ointment.

The survey consisted of a general part and a key recommendation part. The general part included questions about demographics and professional characteristics. In the second section, GPs rated their agreement with 21 statements for each key recommendation. One statement was used to measure the extent of adherence regarding the recommendation in practice (‘I follow this recommendation in practice’). The other statements concerned possible barriers to adherence based on an existing framework (Table 2) [15,17]. Following this framework, barriers were grouped into three main groups, knowledge-related, attitude-related and external barriers. Knowledge-related barriers may be caused by a lack of awareness or familiarity with the content of the guideline recommendation. Attitude-related barriers could be further subdivided into barriers related to lack of agreement, lack of applicability, lack of self-efficacy, lack of outcome expectancy, inertia of previous practice and lack of motivation. External barriers can be divided into patient-related factors (i.e. GPs may believe that patients are unable to perform necessary actions), guideline-related factors (i.e. GPs may believe that guideline recommendations are too complex) and environmental factors (such as lack of time). A 5-point Likert scale (ranging from ‘Strongly disagree, somewhat disagree, neither agree nor disagree, somewhat agree, to strongly agree’) was used to rate the extent of agreement for each statement. The complete survey is included (S3).

**Table 2.** Possible barriers to guideline adherence.

Item number	Barrier	Description
**Knowledge-related barriers**		
2	*Lack of awareness/familiarity:*	GPs may be unaware or unfamiliar with the (exact) content of the guideline recommendation
**Attitude-related barriers**		
*3,4*	*Lack of agreement:*	GPs may disagree with the content or applicability of the guideline recommendation due to perceived lack or inadequate interpretation in general and more specifically to individual patients
*5,6*	*Lack of applicability:*	GPs may disagree with the applicability of the guideline recommendation due to perceived lack or inadequate interpretation in general and more specifically to individual patients
*7*	*Lack of self-efficacy:*	GPs may believe that they are unable to perform the guideline recommendation because they lack knowledge, training or experience
*8*	*Lack of outcome expectancy:*	GPs may believe that adhering to guideline recommendations will not affect patient outcomes
*9,10*	*Inertia of previous practice/lack of motivation:*	GPs may not follow recommendations because of difficulties in changing habits or old routines or lack of motivation
**External barriers**		
*11,12*	*Patient factors:*	GPs may be unable to reconcile patient preferences and demands with guideline recommendations or believe that patients are unable to perform the necessary action
*13-15*	*Guideline factors:*	GPs may believe that the guideline recommendations themselves are unclear or ambiguous, incomplete, or too complex
*16-21*	*Environmental factors:*	GPs may be unable to overcome barriers in their practice environments, such as lack of time/time pressure, lack of resources/materials, organisational constraints within their own practice (e.g. arrangements with assistants), in other organisations (e.g. out of hours services, pharmacies) or between organisations (e.g. cooperation and arrangements with medical specialists) and lack of reimbursement

Table modified from Lugtenberg et al.^(^^10^^)^.

### Analysis

Descriptive statistics were used to describe demographic and professional characteristics. Perceived adherence rates were determined by calculating the proportion of GPs that ‘strongly agree’ or ‘somewhat agree’ to follow the recommendation in practice. Overall guideline adherence was determined by calculating the average perceived adherence of all key recommendations. Positive formulated statements were recoded to analyse perceived barriers, so that higher scores reflect a higher level of perceived barriers. We calculated the proportion of GPs that (somewhat or strongly) agreed that a barrier existed for each key recommendation. Finally, the correlation between perceived adherence and perceived barriers was calculated by a Spearman’s rank correlation to identify barriers that may explain non-adherence for each key recommendation.

### Ethical approval

This study was exempt from the Dutch Medical Research Involving Human Subjects Act, according to the institutional review board of Erasmus MC (MEC-2021-0157).

## Results

In our study, 213 GPs participated, resulting in a response rate of 54.5% and 142 GPs (66.7%) answered all questions.

### GP characteristics

GP characteristics are provided in Table 3. Respondents were equally distributed between sexes (52% female) and predominantly worked as GP partners. Compared to the total population of Dutch GPs, GP partners were slightly overrepresented in our study [18].

**Table 3.** Demographic and professional characteristics of the responding GPs.

Variable	Number (%)	Total population of Dutch GPs^(^^18^^)^ (%)
**Sex**, n (%)		
Male	94 (47.2)	46.2
Female	104 (52.3)	53.8
Other	1 (0.5)	
**Age**, mean (SD)	46.3 (11.6)	48
**Age groups**, *n* (%)		
<35	40 (20.2)	10.5
35-44	47 (23.7)	30.2
45-54	57 (28.8)	28.6
55-64	49 (24.7)	27.8
≥65	5 (2.5)	2.8
**Type of physician**, *n* (%)		
Independent (GP partner)	130 (65.3)	53.7
GP working for another GP (salaried GP)	11 (5.6)	15.3
Flexible (locum GP)	16 (8.1)	16.1
Other	17 (8.6)	–
In training	33 (16.8)	14.9
**Years working of experience as GP**, mean (SD)	18.1 (9.3)	
**Years working of experience categorised**, n (%)		
<3	7 (4.2)	
4-6	15 (9.1)	
7-9	9 (5.5)	
≥10	134 (81.2)	
**Years working as resident**, mean (SD)	2.1 (1.0)	
**Years working as resident categorised**, *n* (%)		
<3	22 (68.8)	
≥3	10 (31.3)	
**Weekly eczema consultations**, mean (SD)	4.5 (3.1)	
**Weekly eczema consultations categorised**, *n* (%)		
<3	118 (59.6)	
5-9	49 (24.7)	
≥10	31 (15.7)	

GP: General practitioner, SD: Standard deviation.

### Overall perceived adherence and association with barriers

**Figure 1.**
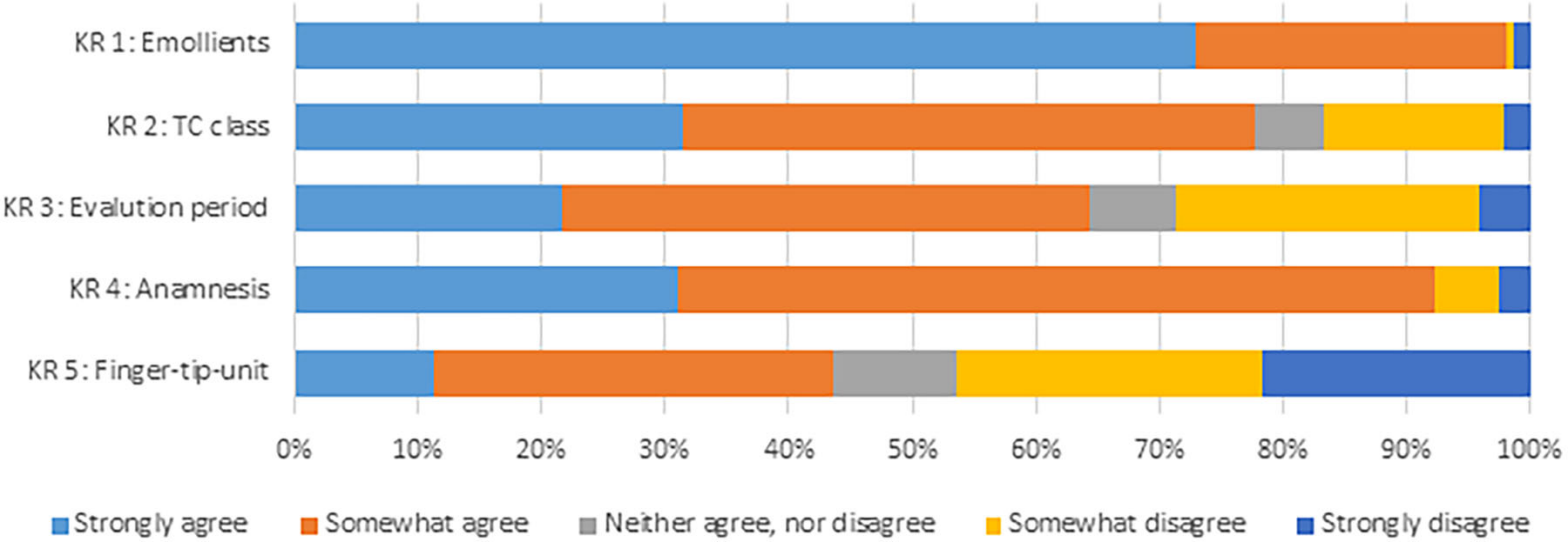
GP perceived adherence to key guideline recommendations.

Mean perceived adherence rate across all key recommendations of the AD guideline was 75.2% (SD: 22.0) (Table 4). Figure 1 provides an overview of the degree of adherence for each recommendation. Perceived adherence varied strongly between key recommendations. Adherence was high on recommendations using emollients in patients with AD (98.1%) and on the performance of a comprehensive anamnesis (92.3%). GPs reported lower adherence (43.7%) for the recommendation on the application instructions on TCS (including the fingertip-unit method). For the perceived barriers, an overview of the percentage of the GPs that agrees that specific barriers apply to key recommendations is provided in Table 4. Across all key recommendations, barriers related to patient behaviour (65.6%), patient preferences (31.5%), and lack of applicability of the recommendation to specific patient groups (29.5%) were agreed upon most frequently. Disagreement with the content of a key recommendation was mentioned the fewest (6.6%; SD 5.6) by GPs as barrier to recommendation adherence. Overall, the correlation between adherence and barriers was strongest for knowledge-related (ρ .55; SD .10), attitude-related, e.g. lack of agreement with the content of a recommendation (range: ρ .40--.62), and guideline factors, e.g. lack of up-to-dateness of a recommendation (range: ρ .42--.53), Table 5. Patient factors were barriers with the weakest correlation with perceived adherence (range: ρ .07--.26). However, the correlation between adherence and barriers varied for each key recommendation.

**Table 4.** Percentage of GPs that report to adhere to guideline recommendations and perceive various types of barriers.

Item number	Description	KR 1: emollients	KR 2: TCS potency class	KR 3: evaluation period	KR 4: anamnesis	KR 5: TCS instructions	Overall (all KRs)	
							mean %	(SD)
**Percentage of GPs that report to adhere to AD guideline recommendations**								
1	General adherence	98.1	77.6	64.3	92.3	43.7	75.2	22.0
**Percentage of GPs that perceive a certain barrier**								
**Knowledge-related barriers**								
2	Lack of awareness/familiarity	3.1	17.9	7.0	5.2	22.5	11.1	8.5
**Attitude-related barriers**								
*Lack of agreement*								
3	Lack of agreement with content: general	2.5	4.7	16.1	2.6	7.0	6.6	5.6
4	Lack of agreement with content: certain parts	8.2	11.8	21.0	3.9	2.1	9.4	7.5
5	Lack of applicability: general	10.6	14.7	40.6	20.6	51.4	27.6	17.6
6	Lack of applicability: to patient	25.2	32.2	46.2	18.7	25.4	29.5	10.5
7	Lack of self-efficacy	3.8	11.2	2.1	3.2	21.8	7	8.3
8	Lack of outcome expectancy	0.6	17	14.7	5.2	11.0	8.5	5.6
9	Lack of motivation	3.9	8.3	19.6	2.6	18.3	10.5	8.0
10	Inertia previous practice	5.1	22.4	25.9	15.5	40.8	21.9	13.2
**External barriers**								
*Patient factors*								
11	Patient preferences	43.6	37.1	43.7	11.3	22.0	31.5	14.4
12	Patient ability/behaviour	80.9	64.3	66.2	48.3	68.1	65.6	11.6
*Guideline factors*								
13	Lack of clarity	2.5	7.7	7.0	5.2	26.1	9.7	9.4
14	Lack of up-to-dateness	2.5	2.8	19.6	7.7	8.5	8.2	6.9
15	Complexity	5.7	6.3	22.4	12.3	36.6	16.7	13.0
*Environmental factors*								
16	Lack of time/ time pressure	5.8	10.5	46.8	30.0	40.1	26.6	18.0
17	Lack of resources/ materials	3.2	3.5	2.1	0.7	9.5	3.8	3.4
18	Organisational constraints	5.2	1.4	27.8	7.3	7.2	9.8	10.4
19	Contradictory with policy of other HCPs	7.7	12.0	7.9	8.7	10.0	9.3	1.8
20	Conflicts in cooperation with other HCPs	6.4	12.0	7.9	8.6	12.9	9.6	2.8
21	Lack of reimbursement	5.1	2.1	5.0	3.3	13.7	5.8	4.6

Percentages represent the portion of general practitioners that agrees or somewhat agrees to adhere to the key recommendation and perceived barrier to adherence. KR: key recommendation, TCS: topical corticosteroids, SD: standard deviation, HCP: healthcare professionals.

**Table 5.** Correlation between GP perceived degree of adherence and perceived extent of various barriers.

Item number	Description	KR 1: emollients	KR 2: TC potency class	KR 3: evaluation period	KR 4: anamnesis	KR 5: TCS instructions	Overall (all KRs)	
							Mean correlation	(SD)
**Knowledge related barriers, r_s_**								
2	Lack of awareness/familiarity	−.42	−.69	−.47	−.50	−.52	−.52	.10
**Attitude related barriers, r_s_**								
*Lack of agreement*								
3	Lack of agreement with content: general	−.61	−.67	−.68	−.54	−.59	−.62	.06
4	Lack of agreement with content: certain parts	−.44	−.42	−.53	−.45	−.46	−.46	.04
5	Lack of applicability: general	−.53	−.45	−.52	−.51	−.36	−.47	.07
6	Lack of applicability: to patient	−.28	−.31	−.57	−.43	−.42	−.40	.11
7	Lack of self-efficacy	−.46	−.45	−.19	−.39	−.64	−.43	.16
8	Lack of outcome expectancy	−.52	−.59	−.54	−.42	−.58	−.53	.07
9	Lack of motivation	−.53	−.47	−.61	−.51	−.58	−.54	.05
10	Inertia previous practice	−.47	−.44	−.39	−.54	−.60	−.49	.08
**External barriers, r_s_**								
*Patient factors*								
11	Patient preferences	−.13	−.25	−.54	−.11	−.27	−.26	.17
12	Patient ability/behaviour	−.05	−.09	−.07	−.09	−.07	−.07	.02
*Guideline factors*								
13	Lack of clarity	−.51	−.55	−.30	−.33	−.42	−.42	.11
14	Lack of up-to-dateness	−.55	−.41	−.62	−.51	−.56	−.53	.08
15	Complexity	−.52	−.47	−.43	−.58	−.48	−.50	.06
*Environmental factors*								
16	Lack of time/ time pressure	−.35	−.23	−.48	−.53	−.34	−.39	.12
17	Lack of resources/ materials	−.34	−.25	−.08	−.30	−.22	−.24	.10
18	Organisational constraints	−.45	−.22	−.36	−.47	−.30	−.36	.10
19	Contradictory with policy of other HCPs	−.45	−.24	−.18	−.39	−.10	−.27	.15
20	Conflicts in cooperation with other HCPs	−.30	−.21	−.15	−.31	−.03	−.20	.12
21	Lack of reimbursement	−.36	−.29	−.17	−.35	−.22	−.28	.08

KR: key recommendation, TCS: topical corticosteroids, SD: standard deviation, HCP: healthcare professionals. Spearman’s rank correlation represents the correlation between each perceived barrier and adherence. A negative correlation implies that a greater perceived extent of a barrier is associated with less perceived adherence.

### Emollients use

Most GPs (80.9%) perceived patient behaviour as a barrier to recommending emollients. Other perceived barriers were patient preferences (43.6%) and lack of perceived applicability to specific patient groups (25.2%). Investigation of the correlation between perceived adherence and barriers shows a weak correlation between adherence and patient-related barriers (range: ρ .05--.13). The strongest correlation with adherence was found for barriers lack of agreement with the recommendation (ρ .61), lack of general applicability (ρ .53), and guideline-related factors, such as lack of clarity of the guideline recommendation or lack of perceived up-to-dateness (range: ρ .51--.55).

### TCS potency class

Approximately three-quarters (77.6%) of GPs reported adhering to the TC potency class recommendation for patients with severe AD. Further exploration of the underlying barriers shows that many GPs identify patient-related factors (range: 37.1--64.3%) and lack of applicability to individual patient characteristics or specific patient groups (32.2%) as barriers for adherence. Analysis of the correlation between adherence and barriers shows strongest correlations for lack of awareness (ρ .69), lack of agreement (range: ρ .31--.67), and lack of outcome expectancy (ρ .59).

### Evaluation (period) of treatment effect

Roughly two-thirds of GPs (64.3%) report adhering to the recommendation concerning the (short) evaluation period for the evaluation of treatment in patients with AD. In addition to patient-related barriers (range: 43.7--66.2%) and lack of applicability (range: 40.6--46.2%), many GPs perceive lack of time (46.8%) as an important barrier to adherence. However, the correlation between barriers and adherence was strongest for lack of agreement (ρ .53--.68), lack of up-to-dateness (ρ .62), and lack of applicability (ρ .57).

### Anamnesis

Perceived barriers to adhering to the recommendation regarding comprehensive anamnesis were patient behaviour (48.3%), lack of time (30%) and lack of applicability (20.6%). Lack of time (ρ .53) significantly correlated with perceived adherence. Other barriers relating strongly to adherence were attitude-related barriers (range: ρ .39--.54) and complexity of this recommendation (ρ .58).

### TCS instructions

Perceived barriers reported to the recommendation on the application instructions of TCS were barriers related to patient behaviour (68.1%), difficulty with applying this recommendation (51.4%), and inertia of previous practices (40.8%). Additionally, compared to other recommendations GPs more frequently (21.8%) reported lack of knowledge/skill as barrier. Correlation between perceived adherence and barriers was strongest for lack of knowledge (ρ .64), inertia of previous practice (ρ .60) and lack of agreement (ρ .59).

## Discussion

### Main findings

This study assessed Dutch GPs’ adherence to the national AD guideline and found varying adherence rates across key guideline recommendations. Perceived adherence was highest for the recommendation on using emollients and lowest for the application instructions of TCS. GPs reported patient-related factors, i.e. patient behaviour and lack of applicability, most frequently as barriers to adherence. Correlation between perceived barriers and adherence was strongest for knowledge-related and attitude-related barriers. The results of this study suggest that these factors may be important to address when aiming to improve adherence.

### Relation between adherence and barriers

Understanding the relation between adherence and barriers is important to develop interventions to improve adherence. In our study, external barriers, such as assumed patient behaviour, patient preferences and lack of time, are frequently reported by GPs as barriers to adherence. However, correlation between reported adherence and these barriers was weak, suggesting that although many GPs report the existence of these barriers, these factors may, in fact, have limited influence on GPs’ adherence. Similar reported large influence of external barriers as perceived by GPs was also found in the study of Lugtenberg et al. [10]. To some extent, externalisation of GPs may explain these results. GPs may unconsciously attribute non-adherence to factors they cannot or are difficult to influence as a defence mechanism to explain non-adherence to themselves [19]. Our finding supports that internal barriers are strongly correlated with perceived adherence. Improving guideline adherence at GPs level should first focus on internal barriers, increasing knowledge of GPs and changing their attitudes towards recommendations that receive low adherence rates such as the application instructions of TCS or TCS potency class. Nevertheless, it is important to realise that external barriers may also influence overall adherence, which may limit adherence to this recommendation among GPs. Addressing external barriers, for example, by using interventions targeting patients’ beliefs, should therefore not be disregarded when aiming to improve overall adherence and care for patients AD [20].

### Guideline adherence in atopic dermatitis

Compared to all recommendations investigated in this and earlier studies, recommendations on the application instructions TCS received the lowest GP perceived adherence rates [17]. Furthermore, compared to other recommendations, GPs reported high rates of perceived barriers of self-reported lack of knowledge, lack of applicability, lack of self-efficacy and inertia of previous practices. These findings align with other studies that found important gaps in the knowledge and use of TCS among GPs. For example, a quantitative study in the UK found that GPs lacked confidence and knowledge, and a study in Belgium showed that GPs are more anxious for TCS than paediatricians and dermatologists [13,14,21]. Although this study was conducted in the Netherlands, these studies suggest our findings are relevant to other countries. After all, the mechanisms that influence guideline adherence in general and TCS recommendations are similar.

TCS are effective and safe; suboptimal prescription and instructions on the use of TCS lead to low patient adherence, corticophobia and undertreatment [22,23]. Adding to the high volume of AD-related consultations and prescription rates of TCS, non-adherence affects many patients [2]. Therefore, improving guideline adherence among GPs could be a successful intervention to influence treatment success at the societal level [3,7,24].

## Implications

The results of this study emphasise the need for additional investment and empowerment of GPs to improve AD care. Fortunately, several methods exist to empower care professionals and enhance AD care [20,25,26]. A potential intervention is to provide (interactive) education. Interactive education has been shown to increase knowledge, improve beliefs and lower worries about TCS and may therefore reduce internal barriers [20,26]. Additionally, GPs prefer interactive education to improve guideline adherence [27]. External barriers may require a more comprehensive approach. A first step to address patient-related barriers, in addition to earlier mentioned patient-targeted interventions, would be to increase patient involvement in developing guidelines and elaborating recommendations [28]. Second, environmental factors, like lack of time, may be addressed by investigating efficient ways to implement recommendations. In the case of application instructions of TCS, an assistant may help GPs to provide instructions or digital applications could be used to ‘shift tasks’ and reduce time constraints among GPs [29]. Furthermore, close cooperation and alignment with all healthcare providers (i.e. pharmacies, and in case of work-related dermatitis employers and occupational physicians) involved in the care for patients is necessary to improve AD care. Last, guideline adherence is not a goal but should improve the quality of care. Additionally, factors like shared decision-making based on the preferences and experiences of patients and professionals affect the quality of care.

### Strengths and limitations

This is the first study quantifying the adherence and perceived barriers by GPs for the AD guideline. Additionally, we explored for the first time the relationship between barriers and guideline adherence, leading to better insights into the mechanisms that influence adherence. A limitation of our study may be the reduced international generalisability due to differences between healthcare systems or the specific content of the Dutch AD guideline. However, in our study, we evaluated guideline adherence and barriers based on key recommendations that are part of international guidelines [7,24]. Additionally, only limited differences between GPs role to the management of AD exist between the Netherlands and other developed countries, suggesting high generalisability [30]. However, barriers related to cost, such as lack of reimbursement, may give different outcomes in other countries, given the financial structure of the Dutch healthcare system. Finally, due to the quantitative nature of our study and the use of an existing framework to classify the barriers we may have missed some barriers; however, we used a wide range of barriers based on a qualitative study to gain comprehensive insights into factors that could reduce adherence [17].

## Conclusion

GPs’ perceived adherence and barriers vary strongly across AD guideline recommendations. GPs report high adherence to recommendations on emollient use, whereas GPs report lower adherence to recommendations on TCS. While GPs perceive patient-related factors as a potential barrier to adherence, strong correlations between perceived adherence and knowledge and attitude-related barriers suggest addressing these factors when aiming to enhance guideline adherence among GPs.

## Supplementary Material

Supplemental Material
