# Supplementary material for: Perceived adherence and associated barriers to the national atopic dermatitis guideline: A survey among general practitioners
Source: Eur J Gen Pract. 2023 Aug 21;29(1):2242583. doi: 10.1080/13814788.2023.2242583 (PMC10443994; doi:10.1080/13814788.2023.2242583)
Supplement: Supplemental Material [file IGEN_A_2242583_SM8512.docx]

# Supplementary data

## Supplement S1. Dutch guidelines in general practice

Clinical guidelines for general practitioners (GPs) are developed by the Dutch College of General Practitioners (NHG). In the past decades, the NHG has produced over 100 evidence-based guidelines, which cover approximately 80% of health problems in general practice PMID: 22668606. All guidelines are developed using public funding and follow a comprehensive protocol. During development, in addition to GPs, other stakeholders, such as patients, other relevant healthcare professionals, and insurance companies, are involved. All guidelines are regularly updated based on recent evidence. During the development, gaps in knowledge are registered in an online database, which allows the NHG to fund specific projects that address the most urgent questions and issues in general practice PMID: 30474455.

Next, implementation is evaluated using performance indicators derived from these guidelines and regional and national initiatives. The NHG itself focuses on implementation of guidelines by incorporating an implementation plan during development of a guideline. Furthermore, to enhance guideline adherence, NHG has made their guidelines publicly available, including as smartphone application and incorporated in a dedicated platform for patients and professionals (thuisart.nl).

On thuisarts.nl, patients (and professionals) can find disease-specific information (including videos) and medical advice. For atopic dermatitis, thuisarts.nl provides an extensive overview of the cause, symptoms, medical advices, treatment options, tapering schemes and videos on applying topical corticosteroids.

## Supplement Table S2. Overview of the panel

| **Name** | **Background** |
| --- | --- |
| Suzanne Pasmans | Paediatric dermatologist, MD, PhD – involved in the development of the eczema guidelines |
| Marie-Louise Schuttelaar | Dermatologist, MD, PhD – involved in the development of the eczema guidelines |
| Gijs Elshout | General practitioner, MD, PhD |
| Karlijn van Halewijn | Senior GP resident, MD, PhD candidate in atopic dermatitis |
| Aviël Ragamin | Resident (not in training) in dermatology, MD, PhD candidate in atopic dermatitis |
| Renske Schappin | Psychologist, epidemiologist, PhD, assistant professor in dermatology |
| Marjolein Lugtenberg | Psychologist, PhD, senior researcher in dermatology |

## Supplement S3. Survey

**Part I - Demographic and professional characteristics**

1. What is your gender?
   1. Male
   2. Female
   3. Other
2. What is your age?
3. In which setting are you currently working as a GP?
   1. Independent
   2. GP working for other GP
   3. Flexible
   4. Other
   5. In training
4. How many years of experience do you have in your current profession?
5. Please provide an estimation of how many patients with eczema consult you weekly.

**Part II – Guideline adherence and barriers**

**Key recommendations**

1. Indifferent agents (ointments) are the basis of treatment and should be advised even when the eczema is calm.
2. In severe eczema, starting (briefly) with a class 3 ‘potent’ topical corticosteroid (rather than a class 1 ‘mild’’ or 2 ‘moderate’ topical corticosteroid) is preferred at all ages.
3. For all types of eczema, evaluate the effect of treatment after 1-2 weeks
4. Take a comprehensive anamnesis in which you always ask for: Onset and course, localisation, nuisance, previous episodes of eczema, influencing factors, treatment
5. Provide oral and written instructions on applying topical corticosteroids for optimal effect. Instruct patients to use the fingertip-unit method (FTU): a dash of ointment the length of an adult's fingertip, 1 FTU, corresponds to approximately 0.5 grams.

Please indicate the extent to which you agree by choosing the most applicable answer.
[Strongly disagree, somewhat disagree, neither agree nor disagree, somewhat agree, strongly agree]

| **Item** | **Description** | **Statement** |
| --- | --- | --- |
| **Adherence** | | |
| 1 | General adherence | I adhere to this recommendation in practice |
| **Knowledge-related barriers** | | |
| 2 | Lack of awareness/familiarity | I am aware of the existence of this recommendation |
| **Attitude-related barriers** | | |
| *Lack of agreement* | | |
| 3 | Lack of agreement with content: general | I agree with the content of the recommendation |
| 4 | Lack of agreement with content: certain parts | I think certain parts of the recommendations are incorrect |
| 5 | Lack of applicability: general | I find this recommendation difficult to apply in practice |
| 6 | Lack of applicability: to patient | I feel that this recommendation does not adequately account for individual patient characteristics or specific patient groups (e.g., comorbidities) |
| 7 | Lack of self-efficacy | I lack the knowledge and/or skills to apply this recommendation properly |
| 8 | Lack of outcome expectancy | I believe that applying this recommendation will lead to better patient care |
| 9 | Lack of motivation | It is not clear to me why I should follow this recommendation |
| 10 | Inertia previous practice | I have trouble changing my existing routines for applying this recommendation |
| **External-related barriers** | | |
| *Patient factors* | | |
| 11 | Patient preferences | This recommendation reflects poorly on patients' wishes and preferences |
| 12 | Patient ability/behaviour | Patients are sometimes unable to perform the required actions or do not keep the made agreements |
| *Guideline factors* | | |
| 13 | Lack of clarity | I find this recommendation clear and understandable |
| 14 | Lack of up-to-dateness | I think this recommendation is outdated and should be revised |
| 15 | Complexity | I find this recommendation too complex to follow in practice |
| *Environmental factors* | | |
| 16 | Lack of time/ time pressure | Applying this recommendation in practice does not work out for me due to workload and lack of time |
| 17 | Lack of resources/ materials | Due to lack of (adequate) materials or equipment, I find it difficult to work according to this recommendation |
| 18 | Organisational constraints | Organisational issues in my own practice (e.g., opening hours, location, inadequate staffing) complicate the application of this recommendation |
| 19 | Contradictory with policy of other HCPs | Because of the policies of other health care providers or parties (e.g., medical specialist, hospital, GP centre, pharmacy, laboratory), I find it difficult to apply this recommendation |
| 20 | Conflicts in cooperation with other HCPs | Lack of communication, clear division of responsibilities, and/or collaboration with other healthcare providers complicate adherence to this recommendation |
| 21 | Lack of reimbursement | The application of this recommendation involves costs that are prohibitive to me |

## Supplement S4. Selection procedure of key recommendations

First, a panel was formed consisting of two dermatologists, a GP, two physicians/PhD candidates in AD (one of the Department of General Practice and one of the Department of Dermatology), and two psychologists with a background in guideline adherence research (Table S2). During several meetings, the panel achieved consensus on selecting 5 key recommendations of the GP guideline for AD, see Table 1.

During the first meeting, consensus was achieved for the preference of a selection of key recommendations from all domains of the clinical guideline: diagnostics, treatment (non-medical), treatment (medical), evaluation, referral. Next, it was agreed that key recommendations should reflect important content of the guideline and should address issues for which GPs would consult the guideline and challenges in AD. Finally, it was agreed to analyse the entire guideline, instead of only the ‘key messages’ (important remarks and recommendations) defined by the authors of the Dutch Guideline to create a broad list of key recommendations, resulting in a list of 14 key recommendations (which included suitable ‘key messages’, see S5).

During the second meeting, consensus was achieved to include four key recommendations (comprehensive anamnesis, emollients use, TC potency for severe AD, evaluation period). The panel agreed that having more key recommendations was not favourable due to the increased burden for participating GPs. Key recommendations that were considered relevant but were not included for this reason were additional (blood) examination, aim of treatment, TC potency for mild AD, TCS application instruction, pulse therapy and referral of patients that not respond to therapy.

During the third meeting, the precise wording of each of the four key recommendations was finalised. Thereafter, independent GPs of two regions (Rotterdam and Groningen, total n=5) and a representative of the national patient organisation for AD were invited to check if these recommendations accurately reflected the AD guideline. All independent GPs agreed that the recommendations reflected key messages of the guideline; however, two GPs suggested that the wording of key recommendations on using emollients and TC potency selection for severe AD could be presented more clearly. Based on their feedback, some minor adjustments were made. Furthermore, two independent GPs suggested including an additional key recommendation on treating AD. Based on previous meetings two key recommendations were considered, TCS application instructions and pulse therapy for patients with relapsing AD. Both recommendations endorsed support from all members of the panel, however, both GPs expressed a slight preference for the recommendation on pulse therapy, while one dermatologist expressed a preference to include both recommendations. After discussion, the recommendation of TCS application instructions was selected due to two main reasons; 1) there were concerns among panel members on the comprehensibility of the recommendation of pulse therapy 2) incorrect TCS usage was seen as an important reason for therapy failure. Thereafter, the final list of key recommendations was checked for comprehensibility by members of the department of general practice of the Erasmus MC University Medical centre.

## Supplement Table S5. Overview of possible key recommendations

| Item | Recommendation |
| --- | --- |
| Diagnostics | |
| 1 | Ask all patients about the onset and course of the skin symptoms, the localisation of the skin lesions (now and during previous episodes), discomfort (itching, scratching, any disturbance of the patient's and family's sleep), discomfort with daily activities, earlier episodes of (constitutional) eczema and the course and (effect of) treatment, influencing factors (such as bathing, showering, soap use), (self) medication. |
| 2 | Inspect the affected skin and palpate the unaffected skin. |
| 3 | Blood tests (IgE-determination) have no therapeutic consequences for atopic eczema or other eczema types. |
| 4 | Additional research is rarely needed. |
| Non-medical treatment | |
| 5 | Indifferent agents, whether in combination with topical corticosteroids or not, form the basis of eczema treatment. |
| Medical treatment | |
| 6 | The goal of treatment is complete remission of the eczema. |
| 7 | Class 1 or class 2 topical corticosteroids are usually sufficient for moderate eczema. |
| 8 | In severe eczema, starting with a class 3 topical corticosteroid is preferred. |
| 9 | Provide verbal and written instructions on applying topical corticosteroids for optimal effect. Instruct patients to use the fingertip unit (FTU): a dash of ointment the length of an adult's distal index finger, 1 FTU, corresponds to approximately 0.5 grams of ointment. |
| 10 | As eczema improves, corticosteroids are tapered off but indifferent agents are continued. |
| 11 | For frequent recurrences, consider preventive pulse therapy with class 2 corticosteroid 1 dd for 2-4 consecutive days per week, in addition to an indifferent agent 1 dd, or refer. |
| 12 | Topical calcineurine inhibitors, such as tacrolimus and pimecrolimus, are not recommended for treating eczema in general practice. |
| Evaluation | |
| 13 | For all types of eczema, evaluate the effect of treatment after 1-2 weeks |
| Referral | |
| 14 | Refer patients who do not respond adequately to treatment or who fail to corticosteroids. Consider referral to a paediatrician in children (for example, in the presence of other atopic conditions). |
